# Grip Strength Normalized by Lean Body Mass: A Novel Biomarker for Cognitive Health in Older Adults

**DOI:** 10.1002/alz70856_103922

**Published:** 2025-12-26

**Authors:** Dvir Dori, Maliha Chowdhury, Nicole D. Anderson, Brian Tan, Danielle D'Amico, Howard Chertkow

**Affiliations:** ^1^ Rotman Research Institute‐Baycrest, TORONTO, ON, Canada; ^2^ Baycrest Academy for Research and Education, Toronto, ON, Canada; ^3^ The Kimel Family Centre for Brain Health & Wellness, Toronto, ON, Canada; ^4^ Rotman Research Institute, Toronto, ON, Canada; ^5^ Kimel Family Centre for Brain Health and Wellness, Toronto, ON, Canada; ^6^ Rotman Research Institute, Baycrest Health Sciences, Toronto, ON, Canada; ^7^ Kimel Family Centre for Brain Health and Wellness and Anne & Allan Bank Centre for Clinical Research Trials, Baycrest Health Sciences, Toronto, ON, Canada; ^8^ Baycrest and Rotman Research Institute, Toronto, ON, Canada; ^9^ University of Toronto, Toronto, ON, Canada

## Abstract

**Background:**

According to work by Boyle and Bennett and colleagues, about half of cognitive decline in the elderly is not explained by amyloid, tau, or other measurable pathologies. Presumably unmeasured aspects of neuronal and synaptic health are key aspects of causation. Maximal grip strength relies on optimal health and function of all motor unit components, including peripheral neural structures. This study explores the relationship between grip strength normalized by lean body mass (Grip/LBM) and cognitive performance, hypothesizing that Grip/LBM reflects brain general cortical neuronal health and is a stronger predictor of cognition than grip strength or lean body mass alone.

**Method:**

A total of 232 older adults (56 males, 176 females; aged 50–95) with subjective cognitive impairment (SCI) or mild cognitive impairment (MCI) were assessed. Grip strength was measured using a Jamar hand dynamometer, and lean body mass was determined via dual‐energy X‐ray absorptiometry. Cognitive performance was evaluated using the Montreal Cognitive Assessment (MoCA). Correlation and multiple linear regression analyses were conducted to assess the association between Grip/LBM and MoCA scores, controlling for age and sex.

**Result:**

Grip/LBM showed a significant positive correlation with MoCA scores (r=0.286, *p* <0.00001). Multiple regression analysis identified Grip/LBM as a significant predictor of MoCA scores (β=4.69, *p* = 0.001), alongside age (β=−0.089, *p* <0.001) and sex (β=−1.56, *p* <0.001), with the model explaining 22.4% of the variance in cognitive performance. Grip strength alone and lean body mass alone were weaker predictors, with minimal or nonsignificant associations. Sex‐specific analyses revealed a stronger correlation in males (r=0.464) compared to females (r=0.305).

**Conclusion:**

Grip/LBM emerged as a robust predictor of cognitive health, outperforming grip strength or lean body mass alone. These findings suggest that neuromuscular function, as reflected by Grip/LBM, may serve as a surrogate marker for brain neuronal integrity. This metric offers potential as a practical biomarker for cognitive health and a target for interventions aimed at mitigating cognitive decline.

